# Feasibility and validity of a statistical adjustment to reduce self-report bias of height and weight in wave 1 of the Add Health study

**DOI:** 10.1186/s12874-016-0227-y

**Published:** 2016-09-22

**Authors:** Janet M. Liechty, Xuan Bi, Annie Qu

**Affiliations:** 1 School of Social Work, University of Illinois at Urbana-Champaign, 1010 W. Nevada, MC-082, Urbana, IL 61801 USA; 2 College of Medicine, University of Illinois, 506 S. Mathews, Urbana, IL 61801 USA; 3 Department of Statistics, University of Illinois at Urbana-Champaign, 725 S. Wright, Champaign, IL 61820 USA; 4 Illinois Statistical Office, University of Illinois at Urbana-Champaign, 725 S. Wright, Champaign, IL 61820 USA

**Keywords:** Self-reported weight, Self-report bias, Statistical adjustment, Statistical correction, Obesity, Overweight, Add Health, Body mass index, Self-report vs. measured, Epidemiology

## Abstract

**Background:**

Bias in adolescent self-reported height and weight is well documented. Given the importance and widespread use of the National Longitudinal Study of Adolescent to Adult Health (Add Health) data for obesity research, we developed and tested the feasibility and validity of an empirically derived statistical correction for self-report bias in wave 1 (W1) of Add Health, a large panel study in the United States.

**Methods:**

Participants in grades 7–12 with complete height and weight data at W1 were included (*n* = 20,175). We used measured and self-reported (SR) height and weight and relevant biopsychosocial factors from wave 2 (W2) of Add Health (*n* = 14,190) to identify sources of bias and derive the most efficient sex-specific estimates of corrected height and weight. Measured, SR, and corrected W2 BMI values were calculated and compared, including sensitivity and specificity. Final correction equations were applied to W1.

**Results:**

After correction, weight status misclassification rates among those who underestimated their weight status were reduced from 6.6 to 5.7 % for males and from 8.0 to 5.6 % for females compared to self-report; and the correlation between SR and measured BMI in W2 increased slightly from 0.92 to 0.93. Among females, correction procedures resulted in a 3.4 % increase in sensitivity to detect overweight/obesity (BMI ≥ 25) and 5.9 % increase in sensitivity for obesity (BMI ≥ 30).

**Conclusions:**

Findings suggest that application of the proposed statistical corrections can reduce bias of self-report height and weight in W1 of the Add Health data and may be useful in some analyses. In particular, the corrected BMI values improve sensitivity --the ability to detect a true positive—for overweight/obesity among females, which addresses a major concern about self-report bias in obesity research. However, the correction does not improve sensitivity to identify underweight or healthy weight adolescents and so should be applied selectively based on research questions.

## Background

As a simple ratio of weight to height (kg/m^2^), body mass index (BMI) is a cost-effective and widely used metric for assessing overweight and obesity [1], despite its limitations as an indicator of excess adiposity or cardiometabolic risk for disease [2]. Self-reported (SR) height and weight are often used in population surveys when direct measurement is cost prohibitive, or when obesity is not the primary focus of study, and have been found to have acceptable validity [3]. However, concerns about the validity of self-reported height and weight among adolescents remain, especially when BMI and weight status are the main outcomes of interest. Researchers have consistently noted that SR height and weight lead to underreporting of obesity prevalence [4–6].

Objective measurement of height and weight using a standardized protocol is more reliable than self-report and should be incorporated in study designs whenever possible [5]. However, large cohort studies that began data collection prior to the rapid rise in obesity --and that have since increased the rigor of biometric measurement – often contain information not available elsewhere. An example of valuable self-reported height and weight data is in the first wave of the National Longitudinal Study of Adolescent to Adult Health (Add Health). Add Health is the largest on-going national cohort study of adolescents originally enrolled in US public schools in 1995–1996 (*n* = 20,774) [7]. It offers a rich source of information and potential knowledge about psychosocial and biological risk and resilience related to weight trajectories, weight loss behaviors, obesity, cardiovascular health, diabetes risk, and metabolic syndrome. Although both measured and self-reported data were collected in Waves 2, 3, and 4, Wave 1 (W1) contains only self-reported height and weight data. This limitation has plagued obesity researchers because of the pressing need for longitudinal analyses of these types of unique cohort data collected prior to the rapid rise in obesity in the US. The foresight of the Add Health study team to begin collecting *measured* height and weight in W2, and yet to also continue collecting self-reported data for comparisons, provide unique opportunities to study the relationship between measured and self-reported height and weight that have not yet been fully exploited.

Several prior studies have examined the validity of self-reported height and weight in the Add Health data [3, 8] but none have proposed a statistical correction. For example, using W2 of the Add Health data, Goodman and colleagues found a high correlation between self-report and measured weight (*r* = .95), height (*r* = .94), and BMI (*r* = .92; *p* < .0005 for all) and the study concluded that self-report height and weight in W1 of the Add Health data were sufficiently valid [3]. Although it found that girls underestimated their weight by an average of 1.02 kg (2.2 pounds) and boys by .19 kg (.4 pounds), no particular recommendations for a statistical adjustment to correct for the observed self-report bias were made at that time [3]. In contrast, statistical corrections of SR bias have been recommended [9] and widely disseminated for many other adolescent data sets [10–12].

Additionally, the high average correlation between self-report and measured BMI among a large sample of adolescents may be falsely reassuring – it may cover up validity issues among known subgroups for whom the correlations between measured and self-report data are higher or lower than average. For example, numerous studies have found that overall, self-report tends to overestimate height and underestimate weight relative to measured values, and weight underestimation is highest among those with overweight or obesity [1, 4, 9]. Given the increasing public health importance of the Add Health data as a source of characterizing obesity trajectories and risk factors over time and the value of using all waves of available data, identifying and testing statistical correction equations for reducing self-report height and weight bias in W1 of Add Health would enable researchers to determine if and under what conditions it may be warranted to apply a correction.

### Purpose of study

We sought to examine the feasibility and validity of a proposed statistical adjustment to correct or mitigate bias in self-reported height and weight in a large, widely used nationally representative panel study of adolescents in the US. Specifically, this study aimed to adjust statistically for the self-report bias in W1 BMI, based on empirical knowledge of the relationship between self-reported and measured BMI at W2. Candidate predictors included demographic, biometric, and psychosocial variables. First (H1), we hypothesized that by using measured and self-reported (SR) height and weight and a wide range of relevant background in wave 2 (W2), it will be feasible to identify efficient regression models to predict measured height and weight at W2 from self-reported values at W2 for males and females.

Second (H2), we hypothesized that the corrected height and weight values at W2 will provide a better estimate of true (i.e., measured) height and weight than SR data alone. Third (H3), when corrected height and weight values are used to calculate BMI percentiles and classify cases by weight status, this will result in a lower rate of misclassification of weight status classification than would SR data alone in W2 among those who underestimated their weight status. Finally, the correction procedures developed from W2 can then be applied to SR height and weight in W1 to generate corrected height, weight, BMI, and weight status at W1. The proposed corrections (H4) are expected to reduce bias in SR height and weight and if so, will mitigate the obesity underreporting bias from self-report that has been documented in the literature.

## Methods

### Data and participants

We used Wave 1 (W1) and Wave 2 (W2) of the National Longitudinal Study of Adolescent Health (Add Health, *n* = 20,745) [13]. Data were collected in 1994-5 (W1) and 1996 (W2; *n* = 14,738); two additional waves of data were collected in 2001–2002 and 2007–2008. Participants were in grades 7–12 at W1. In order to maximize the utility of the proposed procedure for a wide range of Add Health data users, as few exclusion criteria as possible were applied. Participants with complete height and weight data in W1 (*n* = 20,175) as well those with as valid sample weights in W2 (*n* = 14,190) were included. Due to missing values on candidate predictors used for statistical correction, final analytic samples were: W1 (*n* = 19,875) and W2 (*n* = 13,650). Sample characteristics at W1 are shown in Table 1. The use of secondary data was approved and deemed exempt by the Institutional Review Board at the authors’ institution.

**Table 1:** Sample characteristics at Wave 1 among participants with complete self-reported height and weight information at Wave 1, shown by sex

	Total		Female		Male	
Characteristics (range)	N	% or M(SD)	N	% or M(SD)	N	% or M(SD)
Sex			10089	48.6	10086	51.4
Age (12–21 y)		16.0 (1.8)		15.9 (1.8)		16.1 (1.8)
Race/ethnicity						
Hispanic/Latino	3393	11.9	1670	11.8	1723	12.0
NH Black/AA	4497	15.9	2338	16.1	2159	15.7
NH White	10503	67.8	5229	67.7	754	67.8
NH Asian	1439	3.9	685	4.0	5274	3.9
NH other	343	1.7	167	0.4	176	0.6
Parent education						
HS or less	8835	45.1	4425	45.1	4410	45.2
Some college	4039	20.4	2082	21.2	1957	19.6
College or more	6791	31.8	3305	31.0	3486	32.6
Weight status						
Healthy weight	14586	71.9	7552	74.9	7034	69.2
Underweight	640	3.4	282	2.8	358	4.0
Overweight	2819	14.3	1374	14.0	1445	14.6
Obesity	2130	10.3	885	8.3	1245	12.2
Body size estimation						
About right	10470	52.1	4894	49.1	5576	54.9
Underweight	3481	16.9	1207	11.2	2274	22.5
Overweight	6214	30.9	3982	39.7	2232	22.7
Dieting to lose weight	2664	12.7	2059	20.4	605	5.5
Puberty status (1–5)				3.3 (1.1)		3.1 (1.2)
CES-D (0–60)		11.3 (7.8)		12.3 (8.5)		10.4 (7.1)
Self-rated health (1–5)		3.9 (0.9)		3.8 (0.9)		4.0 (0.9)
Self-esteem (1–5)		4.1 (0.6)		4.0 (0.6)		4.2 (0.6)
N=	20175					

Higher scores indicate higher levels of depressive symptoms, self-rated health, and self-esteem. Weight status at Wave 1 is based on body mass index calculated from self-reported height and weight. Sample sizes do not always equal 100 % due to missing values. Sample sizes are shown in raw numbers; percentages are weighted to the US population

*M* Mean, *SD* Standard deviation, *HS* High school, *NH* Non-Hispanic, *AA* African American, *CES-D* Center for Epidemiologic Studies Depression Scale

### Measures

#### Demographics

Age was calculated in months from date of birth and date of interview at W1 and W2. Sex at W2 was used because the variable was cleaned by Add Health staff and is the most complete; all descriptive tables are stratified by sex. Race/ethnicity at W1 was by self-report. Parent highest education was drawn from the parent survey at W1 and missing data were filled in with the adolescent’s report of parents’ highest education at W1. All other candidate predictors were measured at both time points.

#### Height, weight, and body mass index

Self-reported height and weight were collected at both waves. Measured height and weight were also collected at W2. Height and weight were examined separately to obtain predictive regression equations for each variable. Body mass index was calculated by the standard formula (kg/m^2^); for participants under age 20, SAS macros were used to apply the US sex and age adjusted growth charts [14, 15] to obtain age and sex specific BMI percentiles and weight status classifications as follows: underweight (UW < 5th percentile for age and sex); healthy weight (HW = 5th to < 85th percentile); overweight (OW = 85th to < 95^th^ percentile); and obesity (OB > = 95th percentile). For this study, calculations of BMI (or BMI percentile) and weight status assignment were made based on measured height and weight at W2, self-reported height and weight at W1 and W2, and corrected height and weight at W1 and W2.

#### Puberty

Perceived physical maturity was measured with a self-assessment item (range 1–5) of pubertal development among males and females as follows: *How advanced is your physical development compared to other boys/girls your age?* Response set included: *I look… younger than most, younger than some, about average, older than some, older than most.* Although other indicators of puberty were available (e.g., menarche for girls, voice change for boys), subjective physical maturity taps into self-image [16] in that could affect self-reported weight or height, and it provides a common assessment for both sexes.

#### Psychosocial factors

*Body size estimation* was assessed with the following item: *How do you think of yourself in terms of weight?* [very underweight (UW), slightly UW, about right, slightly overweight (OW), very OW]. Very and slightly UW were collapsed, and very and slightly OW were collapsed to yield a three level class variable for body size estimation (UW, About right, OW). Accuracy and inaccuracy of body size estimation were tested for inclusion in the prediction model but were dropped as non-contributing factors. *Depressive symptoms* were assessed at each wave with the Center for Epidemiological Studies Depression Scale (CES-D) [17]. *Self-esteem* was measured with a 6-item adaptation of Rosenberg’s 10-item self-esteem scale scored on a 4-point scale [18]. The 6-item adapted scale was developed by the Add Health research team [19], and has been found to be unidimensional and have good reliability [20]. Sample items were: *You have a lot of good qualities* and *You have a lot to be proud of*. Response categories ranged from 1 to 5 (Strongly Disagree to Strongly Agree), and items were recoded as needed so that higher scores reflected higher self-esteem. *Self-rated health* was assessed with a single item, developed by the original Add Health research team and scored on a 5-point scale: *In general, how is your health? Would you say…* Response categories ranged from 1 to 5 (excellent, very good, good, fair, poor) and were reverse coded so that higher scores reflect greater self-rated health. This item is nearly identical to that used in the Medical Outcomes Study 36-item Short Form (SF-36): “*In general, would you say your health is*” with the same response categories [21]. Dieting to lose weight was assessed with two items. First, all participants were asked, “*Are you trying to lose weight, gain weight, or stay the same weight*?” [Response categories: lose weight, gain weight, stay the same weight, not trying to do anything about weight]. If participants marked that they were trying to “lose weight” or “stay the same weight” then they were asked a follow up question: “*During the past seven days, which of the following things did you do in order to lose weight or to keep from gaining weight?*” Participants then marked yes/no to a list of weight loss strategies, including dieted, exercised, made yourself vomit, took diet pills, or took laxatives in the past 7 days. If participants marked “dieted in the past 7 days,” they were coded as dieting to lose weight.

### Statistical analysis plan

All final analyses were performed using SAS version 9.2. Longitudinal population sample weights and survey parameters were applied to adjust for the complex survey design of Add Health. We exploited the availability of both measured and self-reported height and weight in W2 to develop a statistical correction for the self-reported weight and height in W1. First, we fit the W2 model and conducted model selection; we then applied the W2 model to W1 data to obtain statistically corrected values, as described in more detail below.

In the first stage, we fit two linear regression models with response variables being W2 measured weight and measured height and we used all candidate predictors. Candidate predictors were characteristics known to influence perceived body weight or height, including self-reported height and weight, sex, age, puberty status, race/ethnicity, parent education, depressive symptoms, dieting, body size estimation, self-rated health, and self-esteem at W2. We then used the Akaike Information Criterion (AIC) to select the most efficient predictors of measured height and weight values without overfitting the data. We also tested two-way interactions between all significant predictors for each regression model. Categorical predictors were treated as class variables in order to generate a single regression equation. Final models for weight (model 1) and for height (model 2) are shown in Table 2.

**Table 2:** Final multiple regression models predicting measured height and weight at Wave 2 from self-reported height, weight, and other candidate factors at Wave 2

	Model 1					Model 2				
	Measured weight at wave 2					Measured height at wave 2				
	B	SE	95 % CI		P	B	SE	95 % CI		P
Intercept	8.20	1.47	5.31	11.09	<.0001	9.53	0.27	9.01	10.06	<.0001
SR Height W2						0.85	0.00	0.84	0.86	<.0001
SR Weight W2	0.95	0.00	0.94	0.96	<.0001					
Age in months	-0.01	0.01	-0.02	0.00	0.049	0.00	0.00	0.00	0.00	<.0001
Puberty status	0.22	0.10	0.03	0.41	0.021	0.05	0.01	0.03	0.07	<.0001
Weight status by SR (HW)										
UW	3.67	0.59	2.51	4.83	<.0001	-0.92	0.07	-1.06	-0.79	<.0001
OW	2.46	0.35	1.78	3.14	<.0001	0.17	0.04	0.10	0.25	<.0001
OB	2.86	0.51	1.86	3.87	<.0001	0.40	0.04	0.32	0.49	<.0001
Body size estimation (About right)										
Underweight	-1.76	0.30	-2.35	-1.17	<.0001	0.13	0.04	0.06	0.20	<0.001
Overweight	3.68	0.28	3.13	4.23	<.0001	-0.11	0.03	-0.17	-0.04	0.001
Dieting to lose weight	0.93	0.31	0.32	1.54	0.003	-0.08	0.04	-0.15	-0.01	0.029
Depressive symptoms^b^	-0.04	0.03	-0.10	0.01	0.115	-0.01	0.00	-0.02	-0.01	0.000
Self-rated health status	-0.39	0.12	-0.64	-0.15	0.002	-0.04	0.01	-0.07	-0.01	0.009
Self-esteem^b^	0.35	0.20	-0.04	0.75	0.079	-0.06	0.02	-0.10	-0.01	0.014
Race/ethnicity (White)^a^										
Hispanic/Latino						-0.23	0.04	-0.30	-0.15	<.0001
Black/African American^c^						0.03	0.03	-0.04	0.10	0.365
Asian						-0.29	0.06	-0.41	-0.17	<.0001
Other^c^						0.02	0.16	-0.29	0.34	0.882
Sex (Male)	^a^					0.65	0.03	0.59	0.71	<.0001
Parent education (College degree)	^a^					0.08	0.02	0.03	0.13	0.001
R^2^	0.91					0.89				

Reference groups are shown in parentheses. Design-based sample weights were applied prior to analyses. Two-way interactions were tested for all significant predictors but none were retained as contributing factors based on AIC selection

SR, self-reported; HW, healthy weight; UW, underweight; OW, overweight; OB, obesity

^a^In Model 1, candidate predictors with empty cells were removed during the AIC selection procedure as non-contributing factors

^b^In Model 1, depressive symptoms and self-esteem were not significant alone but were retained during AIC selection as contributing factors

^c^In Model 2, African American and Other race/ethnic categories were not significant (shown above) but were retained during AIC selection because the class variable overall was a contributing factor

In the second stage we applied the final two regression models to W1 SR weight and height in separate analyses. Then, we calculated a corrected BMI score from the corrected height and weight values for each case. We then used the corrected BMI scores to estimate corrected weight status. For adolescents under age 20, we used BMI percentiles derived from the CDC sex and age adjusted growth charts [14] and SAS macro program [15] to calculate BMI percentile and assign weight status. For those 20 years and older, we used the standard formula to calculate BMI [kg/m^2^] and adult cutoffs for weight status.

Finally, we calculated Pearson correlations, sensitivity/specificity, and misclassification rates between measured and corrected weight status among males and females. Misclassification rates are presented in a contingency table comparing weight status based on actual (measured) vs. corrected BMI. In this context, the misclassification rate is defined as the proportion of subjects whose self-reported BMI or corrected BMI does not fall into the same category as the measured BMI. Rates of weight status misclassification after correction among males and among females can be summed overall or across each type of misclassification (over or under estimation) by summing values above or below the diagonal that represents “true” classification (see Table 4). For example, weight status underestimation can be calculated by summing misclassification rates from the upper diagonal of the contingency table (Table 4).

## Results

Findings provided support or partial support for each hypothesis. Our first hypothesis concerned the feasibility of identifying an efficient model for predicting true height and weight in W2. To adjust for bias in self-reported (SR) weight and height, we used measured weight and height at W2 as the response variables, and regressed against candidate predictors (demographic, biometric, and psychosocial) at W2 using linear regressions (Table 2). A range of factors influenced measured weight (Table 2, Model 1) and height (Model 2). After AIC model selection, 9 contributing factors were retained in Model 1 for weight: self-reported weight, age, puberty, weight status, body size estimation, dieting, depressive symptoms, self-rated health, and self-esteem (*R*^2^ = 0.91). Race/ethnicity did not contribute to the efficiency of the prediction of measured weight in Model 1 and so was removed. For predicting measured height at W2 (see Table 2, Model 2), all candidate predictors shown in Table 2 were contributing factors (*R*^2^ = 0.89). In addition, we tested two-way interactions between all significant predictors for each model, but none were retained during the AIC selection process as contributing factors and so are not shown in Table 2.

Our second hypothesis was also supported. We hypothesized that when the above correction procedures were applied to SR data in W2 and used to compute corrected BMI, the corrected BMI values would provide a better estimate of measured BMI than SR data alone at W2. Pairwise Pearson correlations between BMI scores --derived from measured, self-reported, and adjusted height and weight in W2-- were calculated. Before correction, the correlation between self-reported BMI and measured BMI was 0.92 (*P* < 0.0001). After applying the correction procedure, the correlation between measured BMI and corrected BMI increased slightly to 0.93, indicating the proposed correction yielded estimates that were closer to the true values than SR alone.

We also calculated and compared sensitivity and specificity of self-report and corrected BMI in W2 using measured BMI W2 as the reference. Table 3 shows the sensitivity and specificity before and after applying the correction. For males with overweight or obesity (BMI ≥ 25), the sensitivity increased slightly from 87.7 to 88.0 % (<1 % increase); for males with obesity (BMI ≥ 30), the sensitivity increased from 81.1 to 82.9 % (1.8 % increase) with a small loss in specificity. Among females, after correction the sensitivity increased 3.4 % for overweight or obesity, and increased 5.9 % for obesity. In addition, the confidence intervals (SR and corrected) in Table 3 show no overlap among females in the overweight/obesity group but a slight overlap among females in the obesity group. However, non-overlap in confidence intervals may not be a reliable method alone to assess statistical significance [22] because they are heavily influenced by standard deviations. Thus both percent change and confidence intervals should be considered. With the increase in sensitivity after correction, we found small decreases in specificity among females for overweight/obesity group (1.8 %) and obesity group (0.6 %); and these differences are smaller than the percent gains in sensitivity, suggesting an overall improvement of the proposed correction. Since the general concern is that SR bias underestimates incidence of overweight and obesity [1, 6], and there are tradeoffs with sensitivity and specificity, improvement in sensitivity is the most desirable change we would expect when attempting to correct for SR bias in height and weight.

**Table 3:** Sensitivity and specificity of BMI based on self-reported and on proposed corrected height and weight at Wave 2 by sex, using measured values at Wave 2 as the reference standard

	Sensitivity (%) [95 % CI]		Specificity (%) [95 % CI]		Positive Predictive Value [95 % CI]		Negative Predictive Value [95 % CI]	
	SR	Corrected	SR	Corrected	SR	Corrected	SR	Corrected
Male								
OW or OB (BMI ≥25)	0.877 [0.861,0.892]	0.880 [0.864,0.895]	0.954 [0.947,0.959]	0.951 [0.944,0.957]	0.872 [0.856,0.889]	0.866 [0.850,0.882]	0.955 [0.949,0.961]	0.956 [0.950,0.962]
OB (BMI ≥30)	0.811 [0.785,0.836]	0.829 [0.803,0.853]	0.983 [0.979,0.986]	0.978 [0.974,0.982]	0.881 [0.857,0.901]	0.856 [0.831,0.878]	0.971 [0.966,0.975]	0.973 [0.969,0.977]
Female								
OW or OB (BMI ≥25)	0.839 **[0.821,0.856]**	0.873 **[0.857,0.889]**	0.973 **[0.968,0.977]**	0.955 **[0.949,0.960]**	0.911 **[0.896,0.924]**	0.864 **[0.847,0.880]**	0.948 [0.942,0.954]	0.958 [0.952,0.963]
OB (BMI ≥30)	0.738 [0.706,0.769]	0.797 [0.767,0.825]	0.989 [0.986,0.992]	0.983 [0.979,0.986]	0.894 [0.868,0.917]	0.849 [0.820,0.874]	0.969 [0.964,0.973]	0.975 [0.971,0.979]

Proposed statistical adjustments were applied separately to height and weight prior to calculating the corrected BMI. Sensitivity reflects the true positive rate; specificity reflects the true negative rate. Bolded values indicate no overlap between pairs of confidence intervals for SR and corrected BMI

*Abbreviations*: *OW* overweight, *OB* obesity, *BMI* body mass index, *CI* confidence interval, *SR* self-reported

The third hypothesis was that using the proposed corrected SR height and weight to calculate a corrected BMI would result in a lower rate of misclassification of weight status than would SR data alone in W2 among those who underestimated their weight status, and this was partly supported. As shown in Table 4, overall the percentage of misclassification for each weight status category was small after applying the correction. Using measured BMI as the standard, the cumulative misclassification rate after the correction among underestimators was reduced to 5.7 % for males and 5.6 % for females, which was lower than the misclassification rate based on SR alone (6.6 % and 8.0 %, respectively, not shown).

**Table 4:** Weight status classification comparisons between measured and corrected BMI by sex at Wave 2

	Measured BMI weight status W2 (%)				
Corrected BMI weight status W2	UW	HW	OW	OB	Total
Male					
UW	**1.3**	*0.6*	*0.0*	*0.0*	1.9
HW	2.9	**64.9**	*2.8*	*0.3*	71.1
OW	0.0	3.2	**8.7**	*2.0*	13.8
OB	0.1	0.3	1.5	**11.3**	13.2
Total (*n* = 6717)	4.3	69.1	13.0	13.6	100.0
Female					
UW	**0.9**	*0.5*	*0.0*	*0.0*	1.4
HW	3.1	**67.3**	*2.9*	*0.2*	73.5
OW	0.0	3.2	**9.6**	*2.0*	14.8
OB	0.0	0.2	1.4	**8.7**	10.3
Total (*n* = 6933)	4.0	71.2	13.9	10.9	100.0
*n* = 13,650					

Bolded values on the diagonal represent the percent of cases that were jointly classified in the same weight status category by both methods (measured and corrected BMI). Note that the row totals show the “true” weight status rates. Italicized values above the diagonals represent the weight status misclassification rate among those who underestimated their weight status; such misclassification was a total of 5.7 % for males and 5.6 % for females after correction, obtained by summing misclassification rates above the diagonals for males and for females

*UW* underweight, *HW* healthy weight, *OW* overweight, *OB* obesity, *BMI* body mass index

Finally, our fourth hypothesis was that the correction procedure --derived from empirical prediction models in W2 using both measured and SR data – could be applied to the SR height and weight data in W1 to yield corrected height, weight, BMI, and weight status variables that may reduce the SR bias documented in the literature among adolescents. The correction procedure was applied to yield W1 corrected BMI values and W1 corrected weight status classifications. Since there is no objective reference to W1 true height and weight, only descriptive weight classifications based on W1 SR and corrected BMI are shown in Table 5. As shown, 3 % (*n* = 295) of females who self-reported as HW were reclassified as OW and 1.3 % (*n* = 130) of females who self-reported as OW were reclassified as OB after the correction. Among males, less than 1 % (*n* = 86) were reclassified from HW to OW and 1 % (*n* = 101) of males were reclassified from OW to OB after the correction, thus partially mitigating SR bias observed in W2.

**Table 5:** Weight status classification comparison between BMI based on corrected and self-reported height and weight at Wave 1, shown by sex

	Corrected W1								
Self-reported W1	UW		HW		OW		OB		
	n	%	n	%	n	%	n	%	Total
Male									
UW	**143**	**1.4**	207	2.1	0	0.0	0	0.0	350
HW	90	0.9	**6748**	**68.0**	86	0.9	0	0.0	6924
OW	0	0.0	58	0.6	**1258**	**12.7**	101	1.0	1417
OB	0	0.0	0	0.0	34	0.3	**1193**	**12.0**	1227
Total	233	2.3	7013	70.7	1378	13.9	1294	13.0	9918
Female									
UW	**67**	**0.7**	207	2.1	0	0.0	0	0.0	274
HW	51	0.5	**7099**	**71.3**	295	3.0	0	0.0	7445
OW	0	0.0	18	0.2	**1213**	**12.2**	130	1.3	1361
OB	0	0.0	0	0.0	15	0.2	**862**	**8.7**	877
Total	118	1.2	7324	73.6	1523	15.3	992	10.0	9957
N=									19875

Bolded values on the diagonal represent the the number and percent of cases jointly classified as that weight status by both methods (self-report and corrected self-report BMI)

*UW* underweight, *HW* healthy weight, *OW* overweight, *OB* obesity, *BMI* body mass index

## Discussion

This study examined the feasibility and validity of statistical adjustment methods to reduce bias of self-reported height and weight in Wave 1 of the National Longitudinal Study of Adolescent to Adult Health (Add Health). We described a rigorous statistical approach to mitigate the bias introduced by self-reported height and weight in Wave 1 of the Add Health data through statistical adjustment. Because the Add Health longitudinal study provides a sufficiently large sample size, many candidate predictors, and both self-reported and measured weight and height in W2, we were able to formulate a comprehensive model based on the most salient predictors of discrepancy between self-reported and measured values. We then tested the sensitivity and specificity of the W2 corrected BMI, compared to the W2 true (measured) BMI, and identified the most efficient prediction model to apply to self-reported values at W1.

Although numerous statistical corrections for self-reported height, weight, or BMI have been developed and published for other population data sets among adolescents [11, 12, 23, 24] and adults [25, 26], to date we found none that were recommended for Add Health. It is critical that statistical adjustment models and prediction equation coefficients be dataset specific, or at least country and population specific [24], unless the samples are truly comparable (e.g., nationally representative of same population in the same year).

The statistical correction described here can be used in two ways. First, if a study intends to draw from the full W1 sample and overweight or obesity are the outcomes of interest, the equations and coefficients (Table 2) can be applied as described. Second, if an obesity analysis is planned that will draw upon a smaller, distinctive analytic sample, such as foster youth or youth with same-sex partners, then the *procedures* outlined here for building, testing, and applying sex-specific prediction models to obtain corrected BMI scores can be followed but tailored to a particular subpopulation in Add Health. Finally, studies based on other sources of data can use these general principles and procedures to develop BMI corrections for other datasets.

A limitation of the study is that there was a one year difference between W1 and W2, so although the prediction models were based on W2, it is possible that factors influencing self-reported height and weight at W1 and W2 were not the same. Another limitation was missing data: the W2 correction equation could only be built upon cases with complete data. Finally, these statistical corrections are conservative and so have a relatively small impact on correcting extreme discrepancies between measured and SR values. Despite limitations, when obesity is modeled as an outcome, applying this statistical correction does appear to reduce SR bias and improve sensitivity for detecting obesity in W1 of the Add Health data, particularly among females. If the outcome of interest is detecting underweight or normal weight only, the correction would offer little advantage.

## Conclusion

In summary, findings in this study address a growing concern about bias in self-reported height and weight among adolescents, specifically the underreporting of obesity. Using unique features of a well-known and widely used US national data set, we developed and tested a formula to statistically adjust for observed self-report bias in height and weight among adolescents, using a wide variety of characteristics to account for how each affects self-report bias on average among male and female adolescents. The resulting corrections were used to compute corrected BMI scores and weight status classification. Corrected values improved sensitivity --the ability to detect a true positive—for obesity among females, which addresses a major concern about self-report bias in obesity research. Corrections did not improve sensitivity to detect underweight or healthy weight in adolescents of either sex, so the correction should be applied selectively, depending on the research questions and outcomes of interest.

## Abbreviations

BMI: Body mass index
HW: Healthy weight
OB: Obesity
OW: Overweight
SR: Self-reported
UW: Underweight
W1: Wave 1
W2: Wave 2
